# Validity of the Connor Davidson Resilience Scale (CD-RISC) in adolescents and young adults in Sweden: a think aloud approach combined with a Rasch analysis

**DOI:** 10.1186/s12955-025-02447-y

**Published:** 2025-10-30

**Authors:** Anna Möllerstrand, Jeanette Winterling, Anders Kottorp, Anna Jervaeus

**Affiliations:** 1 Department of Nursing Science, Sophiahemmet University, Stockholm, Sweden; 2 Department of Neurobiology, Care Sciences and Society, Division of Nursing, Karolinska Institutet, Stockholm, Sweden; 3 ME HHLH, Karolinska Comprehensive Cancer Centre, Karolinska University Hospital, Solna, Sweden; 4 Faculty of Health and Society, Malmö University, Malmö, Sweden

**Keywords:** Resilience, psychological, Connor-Davidson Resilience Scale, Psychometrics, Adolescents, Young adults, Questionnaire

## Abstract

**Background:**

Resilience is defined as the ability to adapt to adversity, most widely assessed with the self-reported questionnaire Connor-Davidson resilience scale (CD-RISC). While previous studies have demonstrated CD-RISC psychometrically sound, it has not yet been validated in a young Swedish population. Therefore, the aim was to evaluate validity evidence based on test content, response processes and internal structure of the Swedish CD-RISC-25 and the 10-item combination among adolescents and young adults.

**Methods:**

This study is divided into two phases. To ensure validity based on test content eight think-aloud interviews were conducted in phase 1. The results guided refinements prior to phase 2. In phase 2, 1500 16–30-year-old individuals, randomly sampled from the general population to participate by completing an online questionnaire. A Rasch rating scale model analysis was performed.

**Results:**

Think-aloud interviews identified difficult wordings in two items, clarified in the survey before phase 2. The Rasch analysis (*n* = 325) indicated that the response categories were well-functioning, and local independence was demonstrated among all items. Six items in CD-RISC-25 and one item in CD-RISC (10 items) displayed misfit to the chosen model. The iterative process resulted in shortened 19- and 9-item versions. Person-response validity and unidimensionality were acceptable for the shortened CD-RISC (9 items). No floor or ceiling effects were detected. Person-separation index showed that both versions could differentiate between three different levels of resilience in the sample. Differential item functioning (DIF) was observed in one item related to gender in CD-RISC (9 items). In shortened CD-RISC-25 DIF was found in five items related to gender and in two items related to age.

**Conclusions:**

When used in a young Swedish population, validity evidence based on test content of CD-RISC is lacking due to difficult wordings and may benefit from additional clarification regarding two items. Shortened versions of CD-RISC (19- and 9-item combinations) demonstrated generally acceptable validity evidence based on response processes and internal structure, but only CD-RISC in the 10-item combination exhibited unidimensionality and met set criteria for person-response validity. For assessing resilience in a young Swedish population, CD-RISC in the 10-item combination appears to be more suitable.

## Background

Despite encountering potentially traumatic events, many individuals continue to experience positive emotions and are not disrupted in their ability to function [1]. This phenomenon highlights the concept of resilience, defined as the capacity to positively adapt or bounce back after adversity [1, 2]. When confronted with traumatic events or other forms of adversity, an individual’s level of resilience appears to significantly influence their mental well-being and quality of life. Resilience is inversely related to depressive symptoms among those who have experienced a natural disaster [3]. In the context of cancer, resilience is associated with satisfaction with life and health-related quality of life [4] and has been found to reduce emotional distress [5–7].

Resilience is conceptualized both as a stable construct, representing a personality trait, and as a dynamic process that evolves over time [5, 8]. Resilience processes can involve social support and strategies like coping and emotion regulation [5, 9]. Resilience might depend on the developmental stage of the individual [10]. Some evidence suggests that age influences resilience levels, with younger participants scoring significantly lower on resilience measures in both community samples [3] and clinical populations [11, 12].

Viewing resilience as a process implies that it can potentially be developed at any time in life [13]. Interventions focusing on resilience may support people affected by severe adversity by decreasing the likelihood of developing stress-induced mental ill-health [2, 14]. This might be especially important for younger populations, as they are still in critical stages of psychological and social development. One example of this is a psychosocial support program targeting resilience for young patients with cancer [15].

To be able to evaluate such interventions, a valid and precise outcome measure targeting resilience is essential. In a review of resilience measurement scales, one of three measures identified as having the best psychometric ratings were the Connor-Davidson Resilience Scale (CD-RISC) [16]. CD-RISC is also the most widely used resilience scale, with translations in 90 languages, including Swedish. The original version consists of 25 items (CD-RISC-25), with two shorter versions also available: a 10-item version (CD-RISC-10) and a 2-item version (CD-RISC-2) [17]. The scale measures resilience, seen as personal and interpersonal traits and resources that help individuals sustain functioning and generate positive emotions and experiences when faced with adversity [18].

While the original validation study of CD-RISC suggested a five-factor model [18], this has proven difficult to replicate, and most subsequent research supports a unidimensional structure [19–21]. Most previous psychometric evaluations of CD-RISC have used factor analysis [17]. Nonetheless, item response theory models, like Rasch analysis, offer several advantages over classical test theory models [22]. The Rasch model is particularly suited for measuring latent traits and is appropriate for ordinal data, as is the case for CD-RISC. Secondly, data distribution may not be normal, as demonstrated in previous research on CD-RISC-25 [21], which is possible to accommodate using the Rasch approach [22]. To the best of our knowledge, only a limited number of Rasch analyses have been performed on CD-RISC internationally. Three studies with samples of young people, have been conducted: one on CD-RISC-25 with Spanish adolescents [23] and two on CD-RISC-10 with university students - one in Australia [24] and the other in both Australia and Canada [25]. The CD-RISC-25 has also been evaluated with the Rasch approach in Spanish non-clinical adults [26, 27].

In Sweden, a previous evaluation of CD-RISC with factor analyses concluded that CD-RISC-25 demonstrated good psychometric properties, including predictive and discriminant validity [21]. However, the target population consisted of 45–84-year-old adults and when using a scale in new contexts and populations, it is important to investigate aspects of validity, including validity related to test content since it may vary across age groups [28, 29]. The present study was warranted due to the lack of prior evaluation of CD-RISC among adolescents and young adults in Sweden, a necessary step to enable its use in clinical practice and research. In the Swedish context, adolescents aged 16 and above have greater autonomy and may be treated within adult healthcare settings, while 30 years may reflect the transition into independent adulthood. Therefore, adolescents and young adults will be defined as 16–30 year olds in this study.

Since too long questionnaires may lead to a lack of concentration or motivation [30], it is important to psychometrically evaluate the differences between the 25- and 10-item combinations to determine which provides stronger evidence of validity as well as usability. Given the many-faceted concept of validity [31], this required a mixed combination of methodologies to meet the objectives. The overall objective of this study was therefore to evaluate validity evidence based on test content, response processes and internal structure in the Swedish version of CD-RISC-25 and the and 10-item combination in the context of adolescents and young adults.

## Methods

The study is divided into two phases. Phase 1 utilized a descriptive qualitative design with a purposive sample, with the aim being to evaluate validity evidence based on test content of CD-RISC-25 and, if necessary, guide adaptations for enhanced validity before the next phase. In phase 2, a cross-sectional design was used. The aim was to evaluate validity evidence based on response processes and internal structure of CD-RISC-25 and the 10-item combination, using a Rasch rating scale modelling approach for polytomous data in a general population sample of 16–30-year-olds. Further to compare 25 and 10 items to investigate which one is most suitable in the target population. The specific research questions are:

- Are the response categories in CD-RISC functioning as intended? (Step 1)
- Does CD-RISC demonstrate satisfactory local independence and internal scale validity? (Step 2a-b)
- Is there evidence that CD-RISC measures a unidimensional construct of resilience? (Step 3)
- Is there sufficient evidence of validity in response processes? (Step 4)
- Does CD-RISC enable separation of respondents into distinct groups according to their resilience levels? (Step 5)
- Do CD-RISC items function equivalently across certain demographic subgroups? (Step 6)

### Phase 1

Think-aloud interviews were conducted in line with recommendations for ensuring feasibility and appropriateness of self-reported health outcome measures for children and young people [32]. This method involves participants verbalizing their thoughts as they complete a task [33], with the aim to identify potential concerns, and gather suggestions for improved validity based on test content.

#### Participants

Participants were purposively sampled to ensure variability in age (within the range: 16–30 years), gender and educational background. They were recruited from the research group´s personal networks using the snowball sampling method [34]. Nine individuals were invited to participate; eight accepted and completed the interviews, three of whom were from the interviewer’s (AM) network.

#### Data collection

Interviews lasting 30–60 minutes were conducted between December 2023 and January 2024, via individual online video meetings (*n* = 7) or in person (*n* = 1). Participants were instructed to respond to the questionnaire, read each item aloud, and simultaneously verbalize all their thoughts. The interviewer (AM) took concurrent notes of the verbalized thoughts as well as response behaviour, such as hesitations and self-corrections of responses. At the end, participants were asked follow-up questions regarding items they had reacted to, as well as their overall impressions and opinions.

#### Data analysis

The data obtained from the interviewer´s notes were initially sorted, by the interviewer, in relation to specific items. The entire team then held meetings to identify patterns in the participants perceptions. For items with noted potential problems, changes to enhance validity based on test content were discussed and decided upon by the group—within copyright limits—before the next phase was initiated.

### Phase 2

#### Participants

A non-clinical sample was recruited via Statens personadressregister, SPAR (the Swedish state personal address register), which includes all persons registered as residents in Sweden. To be able to perform a sound validity evaluation including subgroup analyses, the sample size goal was set to *n* = 200–300. Given the generally low response rates of young people to questionnaires in Swedish community samples [35], 1500 randomly selected individuals (50% men, 50% women; 16–30-year-olds; from the whole of Sweden) were approached.

#### Data collection

**Fig. 1:**
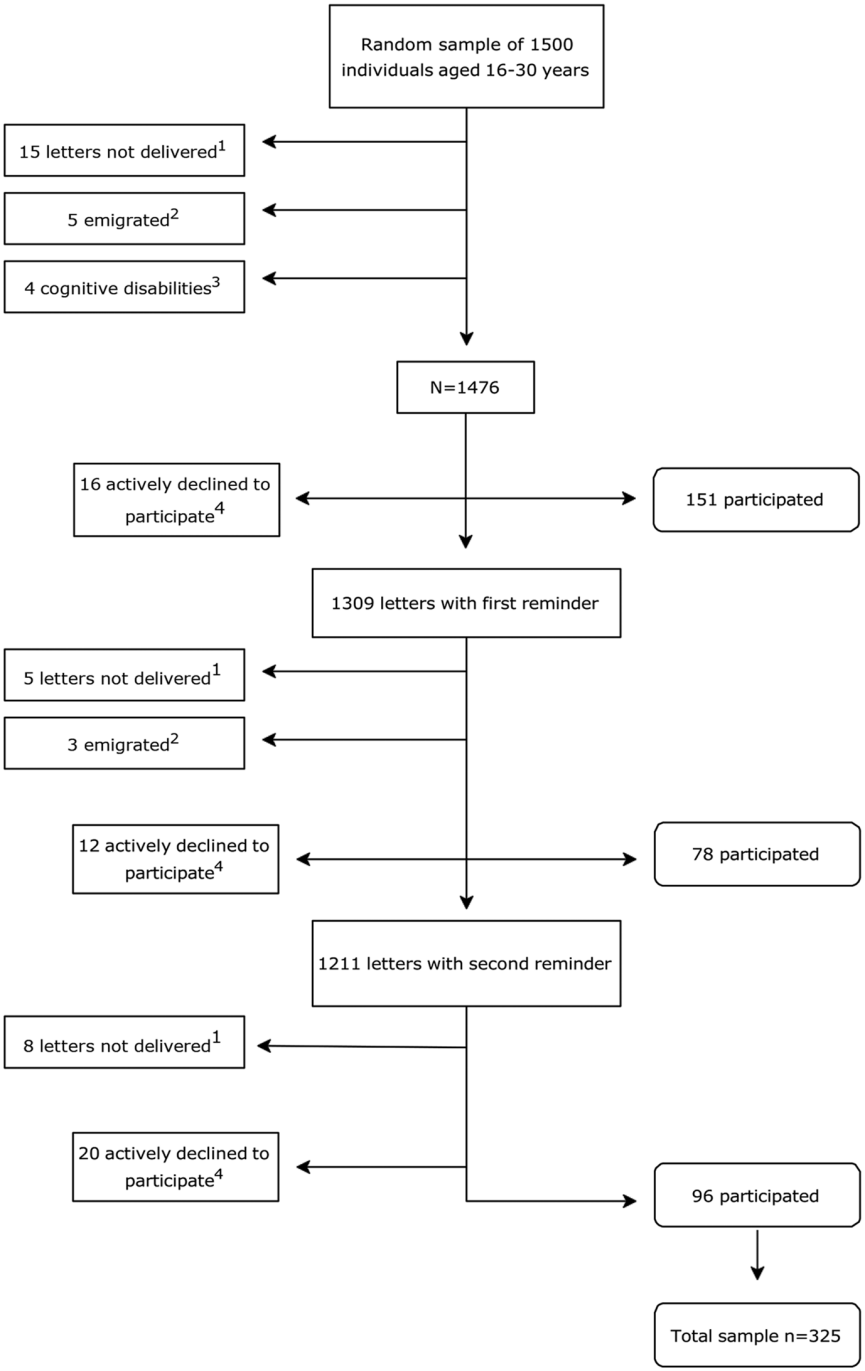
Flowchart of data collection in phase 2. Illustrating distribution of questionnaires and reminders as well as responses received

A letter was sent with study-specific information, a QR-code and web-link for the collection of informed consent and the online questionnaire. The online questionnaire was constructed by an IT-company with extensive experience in research projects. Reminders were sent out twice, approximately two weeks apart. Participants were given the option to answer the questionnaire by telephone interview (one participant requested this). Data was collected between February and May 2024 following the process illustrated in Fig. 1.

#### Measures

The online questionnaire included demographic information and CD-RISC-25. For CD-RISC, the level of agreement on statements is recorded on a 5-point Lickert-scale, ranging from 0-*Not true at all* to 4-*True nearly all the time*. All items share the same polarity, resulting in a score range of 0–40 (CD-RISC-10) and 0–100 (CD-RISC-25), with higher scores indicating greater resilience [17]. CD-RISC was translated into Swedish in 2011, applying a forward- and backward translation process (information based on personal communication with the researchers who performed the translation). However, to the best of our knowledge, neither the translation process nor evaluation of validity based on test content has been published.

#### Data analysis

To calculate descriptive statistics of demographic characteristics and CD-RISC scores, Statistical package for the social science (SPSS® version 29.0.2.0) was used. WINSTEPS® analysis software program (version 5.7.4.0) was used for Rasch analyses. Since all the items in CD-RISC are scored using similar scale categories and are polytomous, a Rasch rating scale model for polytomous data was applied. As outlined below, the Rasch analyses followed a six-step iterative process as employed in previous research [36, 37].

The Rasch modelling approach is a method within item response theory. Rasch analysis examines validity evidence across multiple aspects, including internal structure, response processes, and fairness in testing. Rasch models are suitable for ordinal scales, like CD-RISC, since they transform the ordinal raw scores into equal interval measures by applying a logarithmic conversion of the odds probabilities of responses. Fit statistics are reported as infit and outfit unstandardized mean square (*MnSq*) values, along with standardized fit statistics (*z*-values). The *MnSq* residuals reflect the level of randomness in the data, with a value of 1.0 indicating a perfect fit to the model. *MnSq* values below 1.0 suggest that observations are overly predictable, whereas values above 1.0 indicate greater randomness in the data than anticipated by the Rasch model [38].

*Step 1: Response processes - Rating scale functioning*

Initially, the functioning of the five answer categories was evaluated to ensure that they are ordered logically, with the average measures for each category progressing monotonically. Further, the outfit *MnSq* value for each category should be below 2.0, as outlined by Linacre et al. [39].

*Step 2a: Internal structure—local independence of items*

Local independence was investigated to ensure that each item uniquely contribute to the construct. Item residual correlations was set to ≤0.7, reflecting that the shared variance between the standardized item score residuals should not be > 50% [40].

*Step 2b*: *Internal structure—item goodness-of-fit*

Internal scale validity was also investigated by using the item goodness-of-fit statistics. As sample size has an impact on item fit statistics and our sample size was relatively large (*n* = 325), a stricter criterion for acceptable item goodness-of-fit was set for infit *MnSq* values of 0.7–1.3 logits [41]. Item fit was assessed iteratively, with misfitting items excluded until all items met the goodness-of-fit criterion.

*Step 3: Internal structure—unidimensionality*

To ensure that the scale measures a single construct underling the items, unidimensionality was assessed by performing a principal component analysis (PCA). Unidimensionality was defined as having at least 50% of the raw variance explained by the first latent variable in association with an eigenvalue of ≤ 2 [40].

*Step 4: Response processes—person goodness-of-fit*

Person-fit statistics was then evaluated to assess the extent to which each person’s set of responses corresponded to the model’s prediction. Infit *MnSq* values > 1.4 logits associated with standardized *z* ≥2.0 values were set as the criteria for demonstrating misfit. We accepted that, by chance, up to 5% may show unsatisfactory goodness-of-fit without threatening person-response validity [36]. In contrast with the item goodness-of-fit analysis, participants with unacceptable goodness-of-fit were not excluded, as all respondents were considered relevant for inclusion. Floor and ceiling effects were investigated and considered present if > 15% of the sample scored maximum or minimum respectively [42].

*Step 5: Precision—separation index*

To evaluate the precision of the questionnaire and its ability to detect different levels of resilience in our sample, the person-separation index was calculated. The criterion for the person-separation reliability index was set to ⩾1.5, meaning the scale could separate the sample into at least two different levels of resilience, while > 2 would indicate three levels [40]. To enable comparison with traditional reliability estimates, the Rasch-equivalent Cronbach’s alpha was calculated, with a reliability coefficient criterion set at 0.70–0.95 [42].

*Step 6: Response processes—differential item functioning*

Finally, a differential item functioning (DIF) analysis was conducted since demographic bias can affect measurement accuracy, undermine validity, and influence result interpretation. Differential item functioning was evaluated in relation to age, gender, living arrangements and having children or not. This since resilience might depend on the developmental stage, gender and social context of the individual [10]. The Mantel-Haenszel statistic for polytomous scales was used and considered significant if *p* < 0.01 [40].

## Results

### Phase 1

#### Sample characteristics

Participants were six females and two males with median age 21.5 years. Sample characteristics are summarized in Table 1.

### Validity evidence based on test content

Observations of response behaviours and verbalized thoughts were noted for 11 of the 25 items. Participants were critical or had difficulties understanding the phrasing or meaning of four items (#4,10,13,15). Observations of hesitation and a need to repeat the item were noted for 3 items (#3,11,18). Regarding two items (#8,20), several participants had difficulties understanding certain words. Responding to the item containing a negation (#16) was perceived as challenging. One item (#6) was misinterpreted by a participant. Regarding refinements for enhanced validity based on test content, items could not be rephrased due to copyright restrictions. Nevertheless, for the two items with difficult wording, explanations were added as footnotes before Phase 2. In summary, the results indicated limitations in validity based on test content related to some items. These were earmarked for further examination in the Rasch analysis. See Table 2 for results and refinements made before phase 2.

**Table 1:** Demographic characteristics of sample in phase 1 (*n* = 8)

Age Median: 21.5 years (Range: 17–29)		
**Gender, n (%)**		
Female	6	(75.0)
Male	2	(25.0)
**Highest level of Education**^**1**^**, n (%)**		
Senior high school/Folk high school	5	(62.5)
University	3	(37.5)
**Current occupational status, n (%)**		
Working	3	(37.5)
Studying	3	(37.5)
Studying and working^2^	2	(25.0)
**Native Swedish, n (%)**		
Yes	7	(87.5)
No	1	(12.5)

^1^ Highest level of current/completed education

^2^ Including participants stating *working* and *studying*

**Table 2:** Results of think-aloud interviews, refinements before phase 2 and item misfit to the Rasch model

Item # in CD-RISC-25	Item # in CD-RISC-10	Item	Observations and example quotes	Summary	Refinements before Phase 2	Rasch model misfit
1	1	Adapt to change				
2	-	Close and secure relationships				Misfit in CD-RISC-25
3	-	Belief in fate or God	Gets stuck, repeats question (*n* = 2)	Item might be challenging	None	Misfit in CD-RISC-25
4	2	Can deal with whatever comes	”*It’s an absolute question. It can’t be true that you can handle everything. I definitely don’t agree. If it had said ‘I can handle things that come my way,’ I would have chosen ‘often true.*’” -Part. 4	Item might be challenging	None	
5	-	Confidence from past success				
6	3	See the humorous side of things	“*Is it that you can’t take it seriously, that you laugh it off? I choose ‘rarely true’ because I don’t think you should joke about serious matters*” -Part. 7	Item can be misunderstood	None	Misfit in CD-RISC-10
7	4	Coping with stress strengthens				
8	5	Able to bounce back	Bounce back^1^: Hard to understand (*n* = 2) Hardship^2^: Hard to understand (*n* = 3) “*I don’t understand. Bounce back? Does it mean you bounce back to illness? Hardship -I don’t know what that is*” -Part. 5	Item can be challenging to understand due to wording: *Bounce back* and *Hardships.*	Bounce back explained in footnote: “Recover/return with new strength”^3^ *Hardships* explained in footnote: “Trouble”^4^	
9	-	Things happen for a reason				Misfit in CD-RISC-25
10	-	Give my best effort	“*What is meant here? I don’t get it so I’ll pick the middle option*” -Part. 8	Item might be challenging	None	
11	6	Achieve goals	Repeats question, gets stuck (*n* = 1)	Item might be challenging	None	Misfit in CD-RISC-25
12	-	Does not give up				
13	-	Know where to turn for help	“*Do you mean relatives? Or what do you mean?*” -Part. 1	Item might be challenging	None	Misfit in CD-RISC-25
14	7	Think clearly under pressure				
15	-	Take lead in problem solving	“*I need to read this extra, the formulation was hard*” -Part. 3	Item might be challenging	None	
16	8	Not easily discouraged by failure	Gets stuck, emphasises struggle with negation (*n* = 2) “*I need to read again when it’s a negation and then recalculate what it becomes*” -Part. 5	Item might be challenging due to negation	None	
17	9	See self as strong person				
18	-	Can make difficult decisions	Gets stuck (*n* = 1)	Item might be challenging	None	
19	10	Can handle unpleasant feelings				
20	-	May have to act on a hunch	Hunch^5^: Hard to understand (*n* = 8) “*Not everyone probably knows what the word ‘hunch’ means. It’s kind of like revelation?*” -Part. 6	Item can be challenging to understand due to wording: *Hunch*	Hunch explained in footnote: “Sudden thought or impulse”^6^	
21	-	Sense of purpose				
22	-	Feel in control of life				
23	-	Like challenges				
24	-	Work to attain goals				Misfit in CD-RISC-25
25	-	Take pride in achievements				

Swedish translation in footnotes

^1^Komma igen

^2^Vedermödor

^3^Återhämta sig/återkomma med nya krafter

^4^Besvär

^5^Ingivelse

^6^Plötslig tanke eller impuls

### Phase 2

#### Sample characteristics

325 participants (response rate: 21.7%) consented to participate and completed or partly completed the questionnaire. For sample characteristics, see Table 3.

**Table 3:** Self-reported demographic characteristics and results of CD-RISC for the general population sample in phase 2 (*n* = 321)

	Female (*n* = 200)		Men (*n* = 121)	
**Age (years)**, Median (Range)	24	(16–31)	24	(16–31)
16–17, n (%)	24	(12.0)	19	(15.7)
18–24, n (%)	88	(44.0)	46	(38.0)
25–31, n (%)	87	(43.5)	54	(44.6)
Missing	1	(0.5)	2	(1.7)
**Highest level of education**^**1**^, n (%)				
Elementary school	7	(3.5)	8	(6.6)
Senior high school or Folk high school	115	(57.5)	72	(59.5)
University	77	(38.5)	41	(33.9)
Missing	1	(0.5)		
**Occupational status**, n (%)				
Working	85	(42.5)	59	(48.8)
Studying	71	(35.5)	47	(38.8)
Studying and working^2^	26	(13.0)	4	(3.3)
Sick leave (full- or part-time)^3^	4	(2.0)	3	(2.5)
Unemployed^4^	5	(2.5)	6	(5.0)
Other^5^	6	(3.0)	2	(1.7)
Missing	3	(1.5)		
**Having kids**, n (%)				
Yes	25	(12.5)	13	(10.7)
No	175	(87.5)	108	(89.3)
**Living arrangement**, n (%)				
Alone	31	(15.5)	23	(19)
With someone^6^	169	(84.5)	98	(81)
**CD-RISC score**, Median (Range)				
CD-RISC-25	65.0^7^	(17–96)	68.5^8^	(17–100)
CD-RISC-10	26.0^9^	(3–39)	29.0^10^	(4–40)

^1^ Participants stating gender *other* (*n* = 3) are excluded from this table, *n* = 1 missing

^2^ Highest level of current/completed education

^3^ Including participants stating *working* and *studying*

^4^ Including participants stating *sick leave* only or together with other occupation(s)

^5^ Including participants stating *unemployment* only

^6^ Including participants stating *other* only

^7^ Including participants living with other adult(s) and/or children

^8^*n* = 180, missing *n* = 20

^9^*n* = 106, missing *n* = 15

^10^*n* = 184, missing *n* = 16

^11^*n* = 108, missing *n* = 13

### Rasch analyses

The results of the Rasch Rating scale model analyses are summarized in Table 4.

**Table 4:** Psychometric properties of CD-RISC-25, CD-RISC in the 10-item combination and reduced versions (*N* = 325)

	Original CD-RISC-25 (25 items)	Reduced CD-RISC-25 (19 items)	Original CD-RISC-10 (10 items)	Reduced CD-RISC-10 (9 items)
**Rating scale functioning**	Acceptable	Acceptable	Acceptable	Acceptable
**Local independence**	All items ≤0.7	All items ≤0.7	All items ≤0.7	All items ≤0.7
**Item misfit**	6 Items^1^	None	1 Item^2^	None
**Unidimensionality**	39.6%	41.1%	50.6%	53.7%
Eigenvalue	2.59	1.99	1.54	1.57
**Person misfit**, n (%^3^) Maximum score, n (%) Minimum score, n (%)	31 (9.5) 1 (0.3^4^) 0	22 (6.8) 2 (0.7^5^) 0	20 (6.2) 2 (0.7^6^) 0	16 (4.9) 2 (0.7^7^) 0
**Person-separation reliability** *Person-separation index* *(without extremes)*	2.25	2.24	2.03	2.01
**Person reliability** *Cronbach’s alpha equivalent (KR-20)*	0.97	0.97	0.94	0.94
**Differential item functioning**	4 items^8^ (gender) 1 item^9^ (age) 1 item^10^ (liv.arr.)	5 items^11^ (gender) 2 items^12^ (age)		1 item^13^ (gender)

^1^ Iteration 1: Item 2 *MnSq* 1.94, Item 3 *MnSq* 2.01, Item 9 *MnSq* 1.85, Item 11 *MnSq* 0.58, Item 24 *MnSq* 0.59. Iteration 2: Item 13 *MnSq* 1.41

^2^ Item 3: *MnSq* 1.33

^3^ Calculation of percentage based on *n* = 325

^4^ Calculated percentage based on respondents completing 25 items, *n* = 288

^5^ Calculated percentage based on respondents completing 19 items, *n* = 289

^6^ Calculated percentage based on respondents completing 10 items, *n* = 295

^7^ Calculated percentage based on respondents completing 9 items, *n* = 295

^8^ Item 4 (DIF M women 51.2, DIF M men 47.91, *p* < 0.01), Item 16 (DIF M women 55.91, DIF M men 52.09, *p* < 0.01), Item 23 (DIF M women 52.28, DIF M men 48.28, *p* < 0.01), Item 25 (DIF M women 46.47, DIF M men 49.81, *p* < 0.01)

^9^ Item 12 (DIF M 18–24 y. 49.68, DIF M 25–31 y. 46.4, *p* < 0.01)

^10^ Item 13 (DIF M living alone 53.79, DIF M living with someone 48.3, *p* < 0.01)

^11^ Item 4 (DIF M women 50.74, DIF M men 47.91, *p* < 0.01), Item 15 (DIF M women 50.33, DIF M men 56.09, *p* < 0.001), Item 16 (DIF M women 56.3, DIF M men 52.63, *p* < 0.01), Item 21 (DIF M women 48.57, DIF M men 52.71, *p* < 0.01), Item 25 (DIF M women 45.39, DIF M men 50.01, *p* < 0.001)

^12^ Item 4: (DIF M 18–24 y. 51.41, DIF M 25–31 y. 47.38, *p* < 0.01), Item 21 (DIF M 18–24 y. 47.92, DIF M 25–31 y. 52.01, *p* < 0.01)

^13^ Item 17 (DIF M women 48.45, DIF M men 50.67, *p* < 0.01)

*Step 1: Rating scale response process*

In both the 10- and 25-item combinations, the average measures for the response categories advanced monotonically with an outfit *MnSq* < 2.0 for all scale steps. This met the set criteria.

*Step 2a: Internal structure—local independence of items*

No item residual correlations exceeded our set criterion of ≤0.7 in any of the CD-RISC versions, confirming that all items met the criterion of local independence. The strongest correlation of 0.44 was found between item #3 and #9 in CD-RISC-25.

*Step 2b: Internal structure—item goodness-of-fit*

In the first analysis of CD-RISC-25, five items (#2,3,9,11,24) did not meet the criteria for goodness-of-fit. When these 5 items were removed from the analysis for the second iteration, item #13 demonstrated misfit. After its removal, the remaining 19 items demonstrated infit *MnSq* values in the acceptable range (0.7–1.3). For CD-RISC in the 10-item combination, the first analysis resulted in item #3 (corresponding #6 in CD-RISC-25) demonstrating misfit. After its removal, the remaining 9 items demonstrated acceptable fit to the model. This resulted in shortened 19- (reduced by 24%) and 9-item (reduced by 10%) versions for the next steps of the analysis.

*Step 3: Internal structure—unidimensionality*

The explained variance was 41.1% with an eigenvalue of 1.99 in the reduced 19-item version, from the above step. In the reduced 9-item version the explained variance was 53.7% with an eigenvalue of 1.57, meaning shortened CD-RISC (9 items) exceeded our set criterion of 50%, while shortened CD-RISC-25 did not.

*Step 4: Response processes—person goodness-of-fit*

**Fig. 2:**
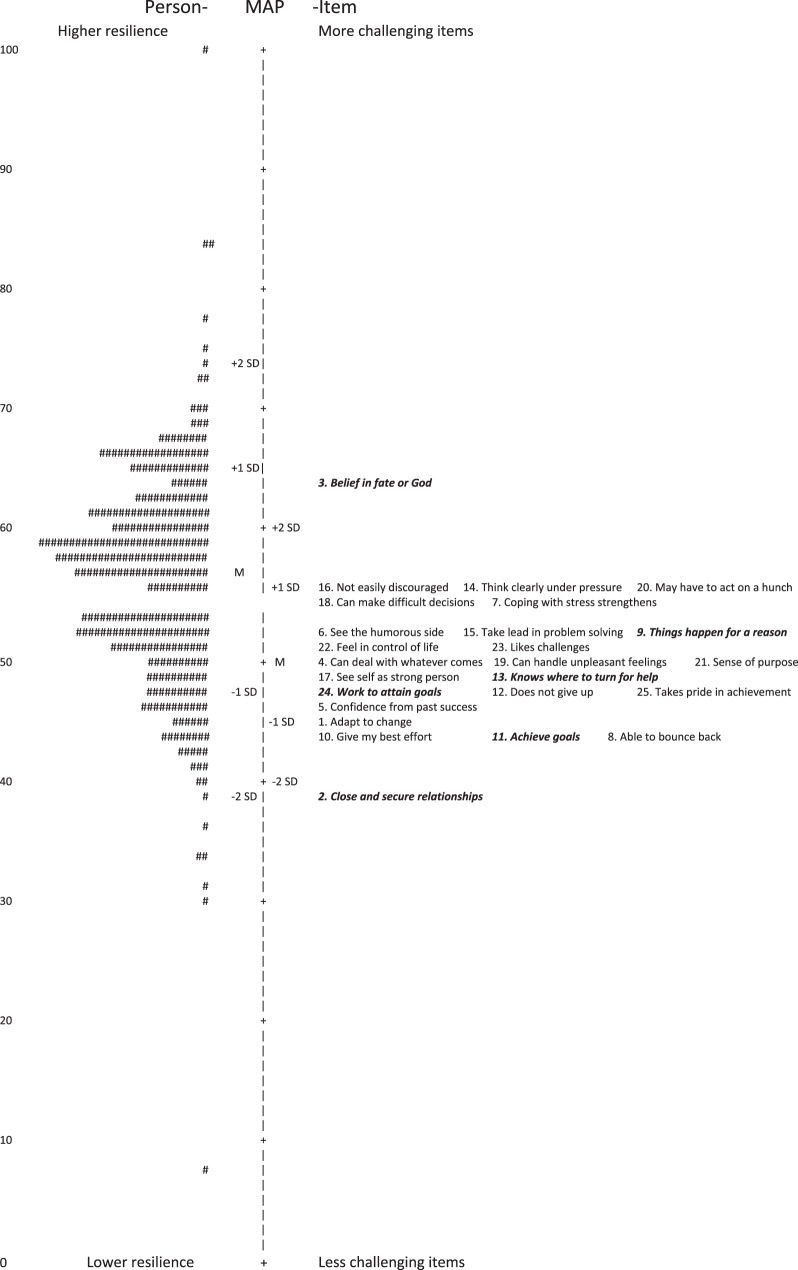
Wright map of CD-RISC-25. To the left: persons where each “#” is one person and displayed by level of resilience, according to the scale on the left. To the right: the 25 items displayed by degree of challenge for each item, according to the scale on the right. Misfitting items in italics and bold

**Fig. 3:**
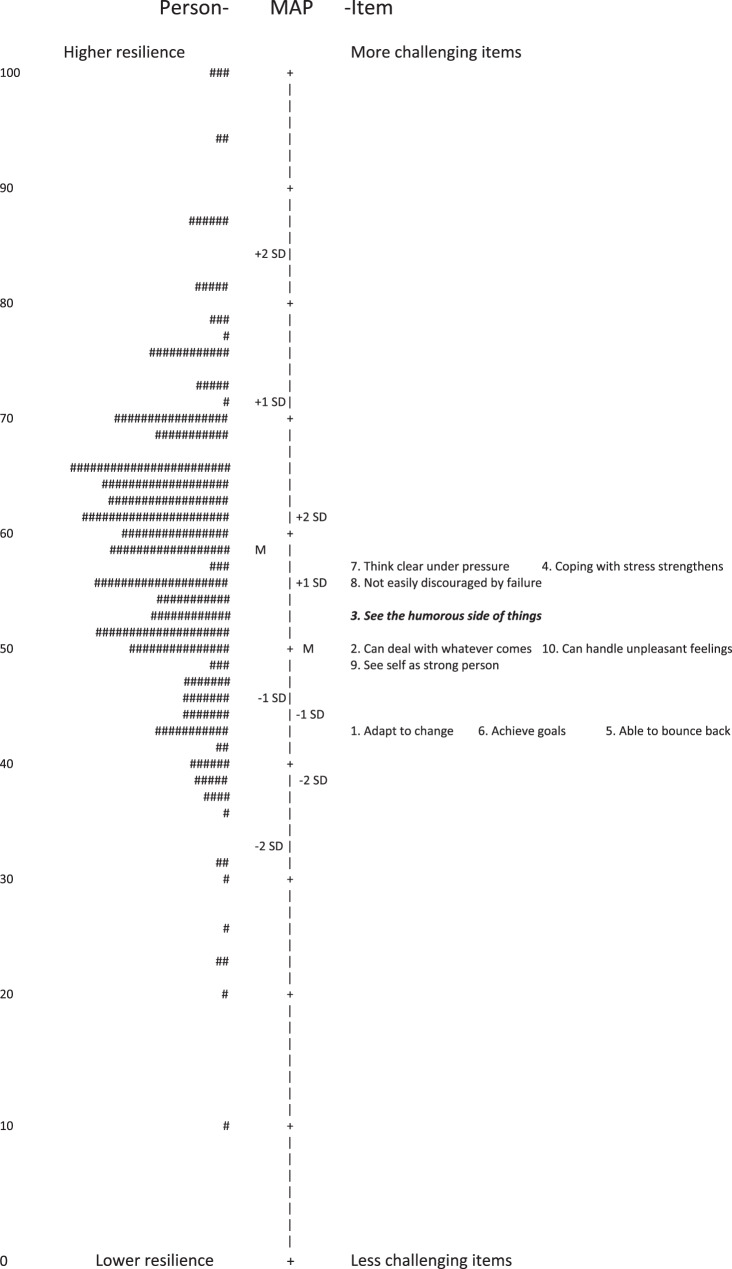
Wright map of CD-RISC 10-item combination. To the left: persons where each “#” is one person displayed by level of resilience, according to the scale on the left. To the right: the 10 items displayed by degree of challenge for each item, according to the scale on the right. Misfitting item in italics and bold

22 respondents in our sample (6.8%) were identified as providing greater variations in responses than expected according to the Rasch model for shortened CD-RISC-25 (19 items). This number was 16 respondents (4.9%) for shortened CD-RISC (9-item combination), meaning that it met the set criteria of < 5%, while shortened CD-RISC-25 (19 items) did not. No floor- or ceiling effects, defined as > 15% of the sample scored maximum or minimum respectively, were detected in any of the versions. Wright maps, shown in Figs. 2 and 3, indicate that the items are perceived as generally easier compared to the resilience levels within the sample. Further, they illustrate that most individuals in the sample fall within a mid-range of resilience and that CD-RISC in the 10-item combination shows a more widely spread distribution of resilience levels compared to CD-RISC-25.

*Step 5. Precision—separation index*

The person-separation index was 2.24 (19-item version) and 2.01 (9-item version). This supports the assumption that both versions of CD-RISC can differentiate between three different levels of resilience. The person reliability coefficient was 0.97 (19-item version) and 0.94 (9-item version), meaning the 9-version met the criterion of 0.7–0.95.

*Step 6: Response processes—differential item functioning*

For shortened CD-RISC-25 (19 items), seven items did not function in an equivalent manner in relation to demographic variables. It was relatively easier for men, compared to women, to agree on two items; #4,16. For three items, #15, 21, 25 it was relatively easier for women to agree. Regarding age, item #4 was relatively easier for 25–31-year-olds compared to 18–25-year-olds, while item #21 was relatively easier for 18–25-year-olds compared to 25–31-year-olds. For shortened CD-RISC (9-item combination) item #17 was relatively easier for women compared to men. In both shortened versions, no significant DIF was identified concerning living arrangements or having children or not.

## Discussion

The aim of this study was to evaluate validity evidence based on test content, response processes and internal structure of the Swedish CD-RISC-25 and the 10-item combination in a sample of adolescents and young adults in Sweden. Results from the think-aloud interviews indicated that, regarding evidence based on test content, the questionnaire could be strengthened for some specific items. The Swedish translation does not seem to fully align with the language usage of a younger Swedish population. Whether this is also true for other age groups in Sweden is not known, since the previous evaluation in an older Swedish population did not address validity based on test content [21]. The additional wording clarifications in phase 2 aimed to enhance validity in relation to test content. The items with clarified wordings (#8, 20) were both found to fit the Rasch model, suggesting that—with the adjustments—they do not seem to pose problems and align with the intended construct of resilience. Although it is not known if these items would misfit the Rasch model in the original translation, there is no evidence that the changes made in formulations regarding test content seem to negatively impact validity in relation to response processes and internal structure.

Overall, the results of the Rasch rating scale model analyses were overall satisfying, with well-functioning response categories, local independence among all items and acceptable item goodness of fit in 19 of 25 items and 9 of 10 items, respectively. No floor- or ceiling effects were detected. However, it is notable, according to the Wright maps, that the items in CD-RISC are better targeted to samples with lower levels of resilience, as also seen in other studies [23, 25, 26, 43]. This is a limitation from a psychometric perspective and renders the questionnaire unsuitable in contexts aiming to identify individuals with high resilience. However, it may be beneficial from a more clinical perspective, as patients with lower level of resilience may be in need for interventions and support and are therefore more important to target.

In terms of unidimensionality and person-response validity, CD-RISC in the 10-item combination outperformed CD-RISC-25. Additionally, using the 10-item combination did not compromise precision, as both versions could differentiate between three levels of resilience in the sample, in line with previous studies in other contexts [27, 44–46]. This is a mathematical concept indicating that the sample can be separated into three distinct groups. However, no clinical conclusions can be drawn about the nature of these groups. The idea that “more items in a scale creates large range of measures as well as more precise measures” is not supported in our findings of the CD-RISC. An in-depth analysis of how items and rating scales empirically are supporting precision in measurement of a unidimensional target construct is crucial to find the optimal version of a tool. Therefore, when evaluating resilience in a young Swedish population—for example, in a clinical setting to evaluate an intervention—CD-RISC in the 10-item combination would be preferable from both a validity perspective as well as a pragmatic patient-burden perspective, described by de Vet et al. [30]. Nevertheless, it might be of interest to obtain a richer picture of resilience in a clinical context, and in such case, CD-RISC-25 would be more appropriate.

The two items (#3,9) displaying the strongest correlation in CD-RISC-25 constitute the factor *spiritual influences*, from the original five-factor model of CD-RISC [18]. Item #3 was perceived as possibly challenging from the results of the think-aloud interviews and both items demonstrated misfit to the model in terms of more randomness than anticipated. This is in line with the previous evaluation in a Swedish population [21] and also seen in other contexts [26, 43]. It supports the assumption that spirituality does not contribute in a systematic way to the concept of resilience in certain populations. The role and impact of spiritual influence on resilience should be further explored in future studies. However, this is not an issue in CD-RISC-10 where the two items are not included.

The results from the DIF analyses imply that gender differences may influence how resilience is expressed or manifested. Results from previous studies in other contexts show variation, with some not detecting DIF regarding gender [23–25, 43], while others do, for single items [26, 27]. However, given the sample sizes in our DIF analyses, our results would need to be explored further before any robust conclusions can be drawn.

The strengths of this study are the mixed method approach used to assess various aspects of validity and the use of an item response theory approach, here with a Rasch analysis, which involved the investigation of validity precision evidence across multiple dimensions. One limitation, however, is that a retest of validity based on test content was not conducted after the refinements in Phase 1. Further, sampling from personal networks might have introduced bias. However, no differences were observed among participants from the interviewer’s network in terms of interview length or the presence of positive or negative comments. When comparing the two CD-RISC versions in Phase 2, a limitation is that they were not administrated separately; the shorter version was extracted from CD-RISC-25. It is unclear whether this could have affected the results, and if so, how. Nevertheless, presenting two similar questionnaires for a study sample to reply to poses ethical challenges, as well as potential threats to validity and reliability. Future studies should investigate the validity of CD-RISC-10 when administrated separately. Although the response rate was considerably higher than that of previous community samples [35], the sample could potentially be biased due to participants’ personal interest, prior knowledge or personal experiences with adversities and resilience. Further, given that the current sample includes individuals aged 16–30, generalizations to younger adolescents should be made with caution.

### Clinical implications

- When using CD-RISC in a young Swedish population, items 8 and 20 could benefit from additional wording clarifications.
- The 10-item combination of CD-RISC seems more suitable towards samples with lower levels of resilience and unsuitable in contexts aiming to identify individuals with high resilience.
- The 10-item combination of CD-RISC is preferable to CD-RISC-25 from both a validity and a pragmatic, patient-burden perspective.
- The findings of this study may serve as a refence sample for comparisons with young populations affected by serious illness.

## Conclusions

This evaluation of CD-RISC indicates that validity based on test content, for some items, is lacking when used in a young Swedish population. This might be addressed with additional clarification of the wording of two items (#8, 20). Further, the psychometric evaluation concludes that after removing 6 misfitting items from CD-RISC-25 and 1 item from the 10-item combination, the validity evidence was generally acceptable, when used in a sample of adolescents and young adults in Sweden. The 10-item combination outperforms CD-RISC-25 in terms of unidimensionality and person-response validity, while maintaining precision. Therefore, the 10-item combination appears to be the more suitable and concise version for assessing resilience among adolescents and young adults in Sweden.

## Data Availability

The datasets used and/or analysed during the current study are available from the corresponding author upon reasonable request.

## Abbreviations

CD-RISC: Connor-Davidsson Resilience Scale
DIF: Differential item functioning
DIF M: Differential item functioning measure
*MnSq*: Mean square
PCA: Principal component analysis
